# Anxious Activists? Examining Immigration Policy Threat, Political Engagement, and Anxiety among College Students with Different Self/Parental Immigration Statuses

**DOI:** 10.1177/00221465241247541

**Published:** 2024-04-29

**Authors:** Erin Manalo-Pedro, Laura E. Enriquez, Jennifer R. Nájera, Annie Ro

**Affiliations:** 1 University of California, Los Angeles, CA, USA; 2 University of California, Irvine, CA, USA; 3 University of California, Riverside, CA, USA

**Keywords:** anxiety, immigrant families, immigration policy, mental health, political engagement

## Abstract

Restrictive immigration policies harm the mental health of undocumented immigrants and their U.S. citizen family members. As a sociopolitical stressor, threat to family due to immigration policy can heighten anxiety, yet it is unclear whether political engagement helps immigrant-origin students to cope. We used a cross-sectional survey of college students from immigrant families (N = 2,511) to investigate whether anxiety symptomatology was associated with perceived threat to family and if political engagement moderated this relationship. We stratified analyses by self/parental immigration statuses—undocumented students, U.S. citizens with undocumented parents, and U.S. citizens with lawfully present parents—to examine family members’ legal vulnerability. Family threat was significantly associated with anxiety; higher levels of political engagement reduced the strength of this relationship. However, this moderation effect was significant only for U.S. citizens with lawfully present parents. These findings emphasize the importance of the family immigration context in shaping individuals’ mental health outcomes.

Prior research has established that undocumented immigrants’ mental health is harmed by restrictive immigration policies and the legal vulnerability they create (Crookes et al. 2022; Crookes, Stanhope, and Suglia 2022; Hatzenbuehler et al. 2017; Philbin et al. 2018; Pinedo and Valdez 2020). Among undocumented college students, deportation threats, academic concerns, future concerns, immigration status, economic insecurity, social exclusion, discrimination, and threat to family are associated with increased stress, anxiety, and depression (Enriquez, Morales Hernandez, and Ro 2018; Flores Morales and Garcia 2021; Manalo-Pedro and Sudhinaraset 2022; Suárez-Orozco and López Hernández 2020; Velarde Pierce et al. 2021). An emerging line of immigration research suggests that policies that threaten to harm undocumented immigrants can also harm the mental health of U.S. citizens who have undocumented family members (Dreby 2015b; Enriquez 2020; Eskenazi et al. 2019; Perreira and Pedroza 2019; Zayas et al. 2023). Yet more research is needed to explore how the family immigration context may affect individual members’ mental health. Specifically, we compare three groups of college students—undocumented students, U.S. citizen students with undocumented parents, and U.S. citizen students with lawfully present parents—to examine the association between their perceived threat to family due to the immigration policy context and anxiety symptomatology.

We also examine one potential buffer in this process: political engagement. The stress process theory suggests that political activism can reduce anxiety by raising one’s sense of control and agency (Thoits 2006). Qualitative research suggests that undocumented students are drawn to immigration-related political engagement in response to hostile policies and have built an immigrant youth movement to challenge policies that harm immigrants and their families (Muñoz 2018; Terriquez and Kwon 2015; Terriquez and Milkman 2021; Wong et al. 2012). U.S. citizens with strong social ties to undocumented family members and friends have also been active in these campaigns for immigrant justice (Enriquez 2014). Undocumented college students who engage in these political actions report feelings of empowerment, safety, and unity, which may partially relieve their worries (Enriquez and Saguy 2016; Nájera 2020). Although political engagement may advance inclusionary immigration-related policies and causes, it is unclear if such political engagement can buffer against poor mental health. Thus, we examine the potential moderation effect of political engagement as a problem-solving coping mechanism on the relationship between immigration-related threat to family and mental health among college students with varying immigration statuses.

## BACKGROUND

### Perceived Threat to Family and Poorer Mental Health

Aligned with the stress process theory, immigration policy is a structural condition that shapes immigrants’ exposure to stressors and, ultimately, their mental health (Ellis 2021; Patler, Hamilton, and Savinar 2021; Philbin et al. 2018; Takeuchi 2016; Thoits 2011). Such policies yield legal vulnerabilities, including economic insecurity, discrimination, social exclusion, and perceived threat to family, which constrain immigrants’ everyday lives, (Ayón, Valencia-Garcia, and Kim 2017; Young and Wallace 2019). Recent studies have demonstrated that these forms of legal vulnerability harm the mental health of undocumented students (Flores Morales and Garcia 2021; Nienhusser and Romandia 2022; Suárez-Orozco and López Hernández 2020; Velarde Pierce et al. 2021). Although most of this work focuses on individual legal vulnerability, we interrogate the broader family immigration context to advance knowledge on how immigration status functions as a structural determinant of health (Montez, Hayward, and Zajacova 2021; Takeuchi 2016).

Perceived threat to family due to the immigration policy context is a legal vulnerability that reflects concerns about how immigration policies compromise the safety of family members and may separate family members across borders; these are related to but distinct from the fear of one’s own deportation (Ayón 2017). This concept captures how deportation threats sow concerns about parental deportation and family separation among both the undocumented and U.S. citizen children of undocumented immigrants (Dreby 2015b; Vega 2023). Thus, although undocumented students may be concerned with their own deportation, their mental health may also be strained by unique fears that an undocumented family member may be deported (Enriquez and Millán 2021; Flores Morales and Garcia 2021). Furthermore, despite not being the direct target of punitive immigration policies or threats, U.S. citizen students with undocumented parents experience immigration-related stressors and poor mental health (Abrego 2019; Dreby 2015a; Hamilton, Patler, and Hale 2019). Indeed, studies have established that parental undocumented status is associated with compromised mental health (Bey and Norton 2021; Landale et al. 2015; Torres and Young 2016). This “multigenerational punishment” (Enriquez 2015) disputes assumptions that second-generation immigrants are immune to immigration-policy-related stressors by virtue of their U.S. citizenship. Thus, undocumented students and U.S. citizen students with undocumented parents likely both experience high perceived threat to family due to the immigration policy context and associated mental health consequences.

Whether immigration-related threat to family also affects U.S. citizen students with lawfully present immigrant parents is unclear. It may be that this group of students perceives little risk because most immigration policy rhetoric focuses on undocumented immigrants. Yet immigration-related threat to family may also stem from federal policies threatening lawfully present residents, even if atypical or hypothetical. The Trump administration, for example, attempted to ban lawful entry (Siddiqi 2019), established an office for denaturalizing U.S. citizen immigrants (Hashemi 2023), and deported refugees (Chhuon, Aleixo, and Solheim 2022). Indeed, the *threat* of a proposed change to “the public charge rule”—which would have made it harder for lawfully present immigrants who used public programs to obtain citizenship—was enough to alter immigrants’ use of public assistance programs (Haley et al. 2020; Miller et al. 2022; Wang et al. 2022). Such rhetoric may stoke concerns regarding the security of lawfully present parents of U.S. citizens.

Thus, attention to whether immigration-related threat to family affects the mental health status of students with varying self and parental immigration statuses is warranted. Incorporating such familial context can offer insight for detecting shared experiences among students from immigrant families, regardless of their own immigration status. Additionally, including groups with varying levels of legal vulnerability can illuminate opportunities for U.S. citizen students with lawfully present parents to leverage their privilege to change immigration policies (García et al. 2021; Link and García 2021). Testing the established relationship between exclusionary immigration policy and poor mental health by self/parental immigration status can reveal the reach of legal vulnerability’s harms and guide future actions to mitigate its consequences.

### “What Do We Do? Stand up, Fight Back!”: Political Engagement as a Moderator

People can reduce the mental health impact of stressful situations by either lowering their exposure or coping with them (Thoits 2010). A robust body of literature has investigated the effectiveness of coping strategies on improving the mental health of young adults (Dijkstra and Homan 2016; Reed-Fitzke 2020; Thoits 2006). However, medical sociology research rarely centers coping mechanisms among immigrant-origin individuals—particularly coping with a devalued, subordinate status (Takeuchi 2016). Notably, universal assumptions regarding stress buffers are not consistently supported when studied among immigrant-origin groups (Sangalang and Gee 2012; Velarde Pierce et al. 2021). Thus, we seek to examine whether political engagement is an effective buffer among immigrant-origin college students.

A commonly understood coping strategy is problem solving, rooted in individuals’ agency or sense of control (Dijkstra and Homan 2016; Reed-Fitzke 2020; Thoits 1994). Because intentional actions (i.e., deliberately setting goals and pursuing them) are beneficial for individuals (Thoits 2006), engaging in any kind of political activism could potentially reduce anxiety. For structurally oppressed groups, dismantling oppressive structural factors may be a “treatment” for poor mental health (French et al. 2020). A recent review on psychology and immigration policy suggests that immigrant-origin students may turn to political engagement as a coping mechanism (Cadenas, Campos, and Minero 2022). That is, the embeddedness of legal systems in the lives of immigrant-origin students may motivate them to challenge these oppressive policies (Negrón-Gonzales 2014). Relatedly, psychological research on marginalized youth engaging in critical action for healing, although relatively new, suggests that there are both developmental benefits associated with youth activism, such as positive identity, intergroup skills, and solidarity across different groups, and potential harms of burnout or disillusionment (Diemer et al. 2021; Maker Castro, Wray-Lake, and Cohen 2022).

Although political engagement as a buffer between immigrant-related stressors and mental health has yet to be investigated, studies examining political engagement as a buffer between race-related stressors and mental health report mixed results. A structural path analysis of economically precarious queer youth found a significant protective effect of activism on health problems among youth of color, but this modest effect was considerably offset by the greater effect of minority stress on health problems (Frost et al. 2019). Among Latinx college freshmen, political activism moderated the positive association between racial-ethnic microaggressions and stress such that the relationship was the weakest for politically active students (Hope et al. 2018). However, political activism did not buffer the effects of racial-ethnic microaggressions on anxiety. It is possible that unmeasured contexts that shape students’ activism, such as one’s immigration status, can clarify which circumstances political engagement protects one’s mental health.

Often, immigrant-origin students become politically engaged to change their material conditions (Muñoz 2018). Although immigrant-origin students may initially be motivated to address numerous causes, like gun control or environmental justice, many direct their activism toward immigrant justice as their political consciousness develops (Enriquez et al. Forthcoming-b; Forenza, Rogers, and Lardier 2017). Upon finding trusted confidants with whom they disclosed their status, undocumented students may feel empowered to organize (Enriquez and Saguy 2016). Qualitative studies have chronicled undocumented students’ advocacy efforts developing undocumented student services on campus (Cisneros and Valdivia 2020; Nájera 2020; Suárez-Orozco et al. 2015), reducing anti-immigrant stigma (Nájera 2015), and championing inclusive policies at the state and federal levels (Abrego 2011; Hinton 2015). Shaped by their family’s unique legal vulnerabilities, however, undocumented students and U.S. citizen students with undocumented parents cautiously weigh whether involvement may endanger themselves and/or their families (Enriquez et al. Forthcoming-b).

Few empirical studies have quantitatively examined immigrant-origin students’ political engagement. Shortly after the passage of Deferred Action for Childhood Arrivals (DACA), a national survey of undocumented students found high levels of political participation and efficacy (Wong, García, and Valdivia 2019). A more recent study on undocumented students in California found more exclusionary perceptions of the immigration policy context increased various forms of political engagement (Rosales, Enriquez, and Nájera 2021). Notably, perceived threat to family had a positive, statistically significant association with political voice (e.g., express a political view during class), collective action (e.g., protest on campus), and individual action (e.g., signed a petition) for undocumented students with DACA or Temporary Protected Status (TPS). However, among undocumented students with no legal status, perceived threat to family was only associated with political voice. Additionally, a recent latent profile analysis on undocumented students’ advocacy communication strategies revealed significant differences in anxiety levels by frequency and method (Cornejo, Ayón, and Enriquez 2023). That is, on average, frequent advocators had higher anxiety scores than infrequent advocators, and students who advocated using social media had higher anxiety scores than those who advocated through organizations. Together, these novel studies suggest that political engagement may vary by one’s legal vulnerability and that political engagement could shape immigrant-origin students’ mental health more broadly.

### Present Study

This study examined the relationship between perceived threat to family due to the immigration policy context and anxiety among college students as a stress process. Building on prior literature on the harms of immigration policy effects on the mental health of undocumented immigrants, we examined perceived threat to family as a stressor among immigrant-origin students with various self/parental immigration statuses. We also investigated whether political engagement—encompassing the dual phenomenon of exercising agency as a problem-solving coping mechanism while taking on the added burden of fighting structural oppression with a subordinate status—buffered the impact of immigration threat on students’ mental health. We asked three research questions:

*Research Question 1*: Does the positive association between threat to family and anxiety symptoms hold among a diverse sample of immigrant-origin college students?
*Research Question 2*: Does political engagement reduce the effect of threat to family on anxiety symptoms among immigrant-origin college students?
*Research Question 3*: Do students with varying self and parental immigration status experience the relationships among threat to family, anxiety symptoms, and political engagement differently?

**Figure 1.**
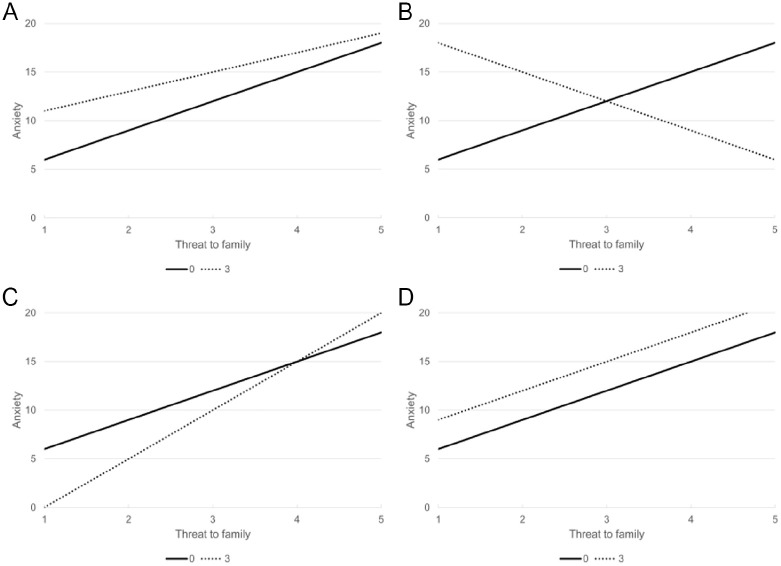
Hypothesized Moderation Effects Overall and by Self/Parental Status. (a) Immigrant-Origin Students (Attenuated). (b) U.S. Citizen Students with Lawfully Present Parents (Protective). (c) U.S. Citizen Students with Undocumented Parents (Exacerbated). (d) Undocumented Students (None). *Note*: We hypothesized that political engagement moderates the association between threat to family and anxiety and that the moderation effect varies by self/parental status.

We expected to detect a positive linear relationship between threat to family and anxiety symptoms. That is, higher mean scores for threat to family would be associated with higher scores for anxiety. We also hypothesized that because of the potential for students to actualize their agency, political engagement would moderate this relationship such that the effect of threat to family on anxiety symptoms would be attenuated among more politically engaged students (Figure 1a).

However, because of varying degrees of legal vulnerability and opportunities for political engagement, we expected differences by self/parental status. We hypothesized that for U.S. citizen students with lawfully present parents (Figure 1b), their concerns may be alleviated through political engagement as an act of exercising agency to solve their problems. Because these students had relatively lower family legal vulnerability and unrestrained freedom to engage politically, we expected the protective effect of political engagement to be the strongest for this group (i.e., inverse relationship between threat to family and anxiety). We also hypothesized that for U.S. citizen students with undocumented parents (Figure 1c), high levels of political engagement would exacerbate anxiety (i.e., intensify the positive relationship between threat to family and anxiety). Although this group of students has the security of their own citizenship, their parents’ legal vulnerability may limit the potential mental health benefits of political engagement. Specifically, they may worry about putting their family at risk while exercising their political voice.

Lastly, we hypothesized that for undocumented students (Figure 1d), high levels of political engagement would not significantly alter the positive relationship between threat to family and anxiety. Given this group’s high individual and family legal vulnerability, the benefits of exercising their agency may not be enough to outweigh their and their family’s reality (i.e., no moderation effect). We analyze U.S. citizen students with undocumented parents as a distinct group from undocumented students because although the exposure—immigration policy threat—is at the family level, the moderator—political engagement—is not.

## DATA AND METHODS

### Data

We used data collected in a survey of University of California (UC) students with immigrant parents between March and June 2020. The overall study assessed the extent to which immigration-related policies produce inequalities in the educational and well-being outcomes of undocumented students, U.S. citizen students with undocumented parents, and U.S. citizen students with lawfully present parents. The eligibility criteria included being over age 18, having at least one immigrant parent, and current enrollment as an undergraduate student. We were unable to develop a randomly generated sampling frame that would allow us to compare across the three self/parental immigration status categories due to a lack of systematically collected and publicly available data at the UC that includes this information. Instead, because the first survey intentionally designed for these three student groups, the team established quotas and corresponding recruitment strategies to ensure each of the comparison groups was sufficient for statistical analysis. For example, after meeting quotas for U.S. citizen students with lawfully present parents, language was revised to specifically recruit students with an undocumented parent. Respondents were recruited from all nine UC undergraduate campuses via emails from each campus’s undocumented student support services office, faculty teaching large general education and ethnic studies courses, and departmental and university newsletters.

The survey was administered via Qualtrics with an estimated completion time of 25 to 35 minutes. Respondents provided informed consent through an information sheet and consent question displayed at the beginning of the survey. Respondents received a $10 electronic gift card as compensation for their time. All project activities were approved by the UC Irvine Institutional Review Board. We collaborated with a community advisory board at each stage of the study design (Enriquez et al. Forthcoming-a).

There were 2,742 respondents: 667 undocumented students, 648 U.S. citizen students with undocumented parents, and 1,427 U.S. citizen students with lawfully present parents. A complete case analysis was performed on 2,511 students whose responses were complete for the variables of interest; no data were missing for self/parental immigration status. Nearly all (93.6%) undocumented students had at least one undocumented parent.

Because part of the data collection overlapped with the initial months of the COVID-19 pandemic, students were asked to indicate what was typical before the pandemic. Although there is a risk of recall bias, multiple studies suggest that college students under current distress recalled negative experiences more accurately than adults (Colombo et al. 2020; Lalande and Bonanno 2011; Wenze, Gunthert, and German 2012). In fact, in supplemental analysis of our data, we did not find higher anxiety scores among respondents who took the survey after the March 2020 pandemic shutdown compared to those who took it before, suggesting that there is not substantial recall bias in this measure (see Appendix A in the online version of the article). Furthermore, the survey included a question that specifically asked the extent to which the COVID-19 pandemic harmed one’s mental health. Analyses of this question suggest that negative mental health effects due to the pandemic did not differ significantly between student groups evaluated in this study (Enriquez et al. 2022; Ro, Rodriguez, and Enriquez 2021).

### Measures

#### Anxiety

Our outcome of interest was anxiety symptomatology, which was assessed using the Generalized Anxiety Disorder-7 (GAD-7), a validated screening tool (Spitzer et al. 2006). Respondents were asked to indicate how frequently (not at all = 0, nearly every day = 3) they were bothered by seven problems during the prior two weeks. Items included “feeling nervous, anxious, or on edge”; “worrying too much about different things”; and “becoming easily annoyed or irritable.” We generated the total anxiety score by summing all seven items for a possible range of 0 to 21.

#### Perceived threat to family

We used the threat to family subscale from the Perceived Immigration Policy Effects Scale (Ayón 2017). The subscale (referred to hereafter as “threat to family”; Cronbach’s α = .93) includes the following three items: Do you worry about the impact immigration policies have on you or your family? Do you fear that you or a family member will be reported to immigration officials? and Do you worry about family separation due to deportation? Responses to each item range from never (1) to always (5), and we calculated an average score.

#### Political engagement

Respondents were asked to indicate how often (never = 0, often = 3) they did each of the following nine actions to express their views: contact a public official—at any level of government—to express your opinion; take part in a protest, march, or demonstration, or rally on campus; take part in a protest, march, or demonstration, or rally off campus; sign a petition regarding an issue or problem that concerns you; boycott a company or product for social or political reasons; buy a certain product or service because you like the social or political values of the company; discuss political issues on social media; wear buttons or display stickers with social or political messages; and express a political point of view during a class discussion (Cronbach’s α = .88). All but the last item was adapted from items in the 2006 National Civic and Political Health Survey (Lopez et al. 2006). Because undocumented immigrants are prohibited from participating in conventional electoral activities, voting behavior was not included in this analysis. We calculated the mean over the nine items; higher scores indicated more frequent political engagement.

#### Self/parental status

Self/parental status was determined by students’ self-report of their own and their parents’ legal statuses. There were three self/parental groups: U.S. citizen students with lawfully present immigrant parents (i.e., legal permanent residents or naturalized citizens), U.S. citizen students with undocumented parents, and undocumented immigrant students. Participants who identified as being born in the United States were classified as U.S. citizens. Participants who identified as being born outside of the United States were asked to identify their current immigration status and given a range of options, including no legal status, DACA, TPS, permanent resident/green card holder, U.S. citizen, valid visa holder (e.g., F1, J1, student visa), and other. A participant was classified as an undocumented student if they occupied a liminally legal status (Menjívar 2006) that included those who selected no legal status, DACA, or TPS or listed a comparable “other” status (e.g., U-visa, asylum seeker); all others born outside the United States were ineligible for the study.

Participants were also asked to identify up to two parents and then asked a series of questions about each, including “Is parent [1/2] a U.S. citizen, permanent resident, or have some other immigration status?” Answer options included no legal status, DACA, TPS, permanent resident/green card holder, U.S. citizen, does not live in the United States, deceased, I don’t know, and other. A participant was classified as having an undocumented parent if one or more parent was reported to have no legal status, DACA, or TPS or participant wrote in an “other” status determined to be undocumented.

#### Covariates

Demographic variables included binary indicators for gender (man/genderqueer = 0, woman = 1) and Latina/o/x race-ethnicity (yes = 1, no = 0). Students were also asked which campus they attended; controlling for campus accounts for differences in local immigration enforcement and access to institutional resources. We also controlled for current year in school (first year, second year, third year, fourth year, fifth year or more, I don’t know).

To control for material conditions, we included family financial strain, food security, and mother’s education. First, we generated a family financial strain score, which was the average of two items on the students’ expectations of how frequently their family will experience bad times and not having basic needs met. The possible range of values was 0 to 4, with higher scores indicative of more financial difficulty. Second, food security status since the start of the academic year was derived from the validated short-form U.S. Household Food Security Survey Module (Economic Research Service 2012). We coded food security scores into three levels: high or marginal food security, low food security, and very low food security. Lastly, mother’s education level was measured as a five-level categorical variable (less than high school, high school or GED, some college, bachelor’s degree, postgraduate degree).

### Analytical Strategy

We ran univariate means and frequency tests to describe the distributions of variables that we used in our multivariate regression analysis. To assess whether there were significant differences by self/parental status, we ran Pearson’s chi-squared tests for independence for categorical variables (i.e., gender, Latinx ethnicity, food security status, mother’s education level, year in school, and campus) and analysis of variance tests for continuous variables (i.e., anxiety, threat to family, political engagement, and family financial strain). Additionally, we calculated Spearman correlation coefficients among anxiety total, threat to family mean, and political engagement mean.

We estimated the focal relationship between threat to family mean (independent variable) and anxiety total (dependent variable) using sequential model building of multivariate ordinary least squares (OLS) linear regressions. The first model assessed the relationship between threat to family and anxiety, controlling for demographics. The second model added political engagement to determine its statistical significance as a predictor of anxiety. The third model tested for moderation by adding an interaction term between threat to family and political engagement. We ran these models first on the full analytical sample and then stratified by self/parental status. As a sensitivity check, we also conducted a three-way interaction between threat to family, political engagement, and self/parental status (see Appendix D in the online version of the article).

To visually illustrate interaction effects by self/parental status, we produced margins plots for the predictive margins and average marginal effects at four levels of political engagement to ease interpretation (never = 0, rarely = 1, sometimes = 2, often = 3). Each plot depicted the average effect of a one-unit change in threat to family on anxiety score with its corresponding 95% confidence interval using the coefficients from Model 3.

## RESULTS

### Descriptive Results

Descriptive statistics are listed in Table 1. The sample was primarily women (75.7%) and Latinx (68.6%). This gender breakdown aligns with extant studies on undocumented college students with majority women samples and consistent with national, state, and institutional trends that indicate that women comprise a larger percentage of undergraduates (Johnson, Payares-Montoya, and Mejia 2023; National Center for Education Statistics 2023; University of California 2023). Year in college was generally spread evenly among the first four years (20.2%–26.8%), although a small percentage (4.4%) were in their fifth year or beyond. The campuses with the most student respondents were UC Riverside (20.9%) and UC Irvine (18.4%). The campuses with the fewest respondents were UC San Diego (5.6%) and UC Davis (8.5%). The average family financial strain was .90 (possible range = 0–4), indicating low family financial strain. However, roughly half (52.3%) indicated either low or very low food security. The most common level of mothers’ education was less than high school (47.5%). The average anxiety total score was 8.65 (possible range = 0–21). The average threat to family mean was 3.01 (possible range = 0–5). The average political engagement mean score was 1.26 (possible range = 0–3).

**Table 1.** Sample Characteristics by Self/Parental Status, UC PromISE Survey (*N* = 2,511).

	U.S. Citizen Students with Lawfully Present Parents*n* = 1,293	U.S. Citizen Students with Undocumented Parents *n* = 609	Undocumented Students *n* = 609	Total *N* = 2,511	*p* Value
Anxiety, mean (SD)	8.15 (5.8)	9.16 (6.0)	9.21 (6.0)	8.65 (5.9)	***
Gender					**
Man/genderqueer	342 (26.5%)	114 (18.7%)	154 (25.3%)	610 (24.3%)	
Woman	951 (73.5%)	495 (81.3%)	455 (74.7%)	1901 (75.7%)	
Latina/o/x					
No	706 (54.6%)	12 (2.0%)	71 (11.7%)	789 (31.4%)	
Yes	587 (45.4%)	597 (98.0%)	538 (88.3%)	1722 (68.6%)	
Family financial strain, mean (SD)	.59 (.9)	1.20 (1.1)	1.27 (1.1)	.90 (1.1)	***
Food security status					***
High or marginal food security	703 (54.4%)	253 (41.5%)	241 (39.6%)	1197 (47.7%)	
Low food security	269 (20.8%)	138 (22.7%)	118 (19.4%)	525 (20.9%)	
Very low food security	321 (24.8%)	218 (35.8%)	250 (41.1%)	789 (31.4%)	
Mother’s education					***
Less than high school	391 (30.2%)	447 (73.4%)	355 (58.3%)	1193 (47.5%)	
High school/GED	277 (21.4%)	110 (18.1%)	134 (22.0%)	521 (20.7%)	
Some college	262 (20.3%)	37 (6.1%)	83 (13.6%)	382 (15.2%)	
Bachelor’s	217 (16.8%)	12 (2.0%)	33 (5.4%)	262 (10.4%)	
Postgraduate degree	146 (11.3%)	3 (.5%)	4 (.7%)	153 (6.1%)	
Current year in school					***
Year 1	307 (23.7%)	159 (26.1%)	108 (17.7%)	574 (22.9%)	
Year 2	293 (22.7%)	117 (19.2%)	98 (16.1%)	508 (20.2%)	
Year 3	331 (25.6%)	165 (27.1%)	178 (29.2%)	674 (26.8%)	
Year 4	318 (24.6%)	139 (22.8%)	186 (30.5%)	643 (25.6%)	
Year 5+	44 (3.4%)	28 (4.6%)	39 (6.4%)	111 (4.4%)	
I don’t know	0 (.0%)	1 (.2%)	0 (.0%)	1 (.0%)	
Campus					***
Berkeley	78 (6.0%)	32 (5.3%)	49 (8.0%)	159 (6.3%)	
Davis	98 (7.6%)	59 (9.7%)	57 (9.4%)	214 (8.5%)	
Irvine	215 (16.6%)	114 (18.7%)	133 (21.8%)	462 (18.4%)	
Los Angeles	148 (11.4%)	75 (12.3%)	77 (12.6%)	300 (11.9%)	
Merced	97 (7.5%)	75 (12.3%)	45 (7.4%)	217 (8.6%)	
Riverside	307 (23.7%)	112 (18.4%)	106 (17.4%)	525 (20.9%)	
Santa Barbara	101 (7.8%)	61 (10.0%)	65 (10.7%)	227 (9.0%)	
Santa Cruz	159 (12.3%)	57 (9.4%)	51 (8.4%)	267 (10.6%)	
San Diego	90 (7.0%)	24 (3.9%)	26 (4.3%)	140 (5.6%)	
Threat to family, mean (SD)	1.99 (1.1)	4.10 (1.0)	4.08 (.9)	3.01 (1.5)	***
Immigration policies	2.38 (1.3)	4.22 (1.1)	4.44 (.9)	3.32 (1.5)	
Immigration officials	1.78 (1.1)	3.83 (1.2)	3.67 (1.2)	2.71 (1.5)	
Family separation	1.86 (1.2)	4.25 (1.0)	4.14 (1.1)	3.00 (1.6)	
Political engagement, mean (SD)	1.19 (.8)	1.42 (.8)	1.26 (.8)	1.26 (.8)	***

*Note*: Self/parental statuses include U.S. citizen with lawfully present parents (1), U.S. citizen with undocumented parents (2), and undocumented students (3). The following *p* values correspond to chi-squared tests of independence for categorical variables (gender, Latinx, food security status, mother’s education level, year in school, and campus) and analysis of variance for continuous variables (anxiety, threat to family, political engagement, and family financial strain).

** *p* < .01, ***p < .001.

Several differences by self/parental status are noteworthy. U.S. citizen students with undocumented parents are more similar to undocumented students than U.S. citizen students with lawfully present parents on multiple items, including anxiety, family financial strain, food security, and threat to family. Most U.S. citizen students with undocumented parents and undocumented students reported being always or often concerned about every item in threat to family. By contrast, threat to family concerned fewer than 20% of U.S. citizen students with lawfully present parents. Additionally, the racial compositions vary significantly by group, with the fewest non-Latina/o/x students in the group of U.S. citizen students with undocumented parents (2.0%; *p* < .001). U.S. citizen students with undocumented parents were the most politically engaged on average (1.23), followed by undocumented students (1.26) and U.S. citizen students with lawfully present parents (1.19; *p* < .001). Notably, the same political activities were reported most often (i.e., signing petitions) and least often (i.e., contacting public officials to express opinion), although exact proportions varied by group. The distribution of responses for each item for anxiety, threat to family, and political engagement are presented by self/parental status in Appendix B in the online version of the article.

Correlations between anxiety, threat to family, political engagement, and self/parental status were positive and statistically significant for each pair (Table 2). Spearman correlation coefficients for anxiety were .26, .20, and .08 for threat to family, political engagement, and self/parental status, respectively (all *p*s < .001). Threat to family was more strongly correlated with self/parental status (r = .65) than political engagement (r = .32; *p* < .001). Political engagement and self/parental status were correlated (r = .06, *p* < .05).

**Table 2.** Spearman Correlations between Anxiety, Perceived Threat to Family, Political Engagement, and Self/Parental Status, UC PromISE Survey (*N* = 2,511).

	(1)	(2)	(3)	(4)
(1) Anxiety	1.00			
(2) Threat to family	.26***	1.00		
(3) Political engagement	.20***	.32***	1.00	
(4) Self/parental status	.08***	.65***	.06**	1.00

*Note*: Self/parental statuses include U.S. citizen with lawfully present parents (1), U.S. citizen with undocumented parents (2), and undocumented students (3).

** *p* < .01, ***p < .001.

### Multivariate OLS Results for Total Sample

Table 3 displays the adjusted OLS regression results for threat to family on anxiety, controlling for demographics. In Model 1, we observed a statistically significant positive relationship such that students who perceived more threat to family had higher anxiety scores. Each one-unit increase in threat to family increased GAD-7 by 1.15 units (95% CI = .91, 1.38).

**Table 3.** Adjusted Ordinary Least Squares Regression Moderation Results for Threat to Family and Anxiety, UC PromISE Survey (*N* = 2,511).

	Model 1		Model 2	
Variable	Coefficient	95% Confidence Interval	Coefficient	95% Confidence Interval
Threat to family	1.15***	.91, 1.38	1.09***	.75, 1.44
Political engagement			.58	−.08, 1.24
Threat to family × Political engagement			−.04	−.23, .15
Self/parental status				
Student with lawfully present parents	Reference		Reference	
Student with undocumented parents	−1.91***	−2.59, 1.22	−1.76***	−2.46, 1.07
Undocumented student	−2.04***	−2.72, 1.36	−1.83***	−2.53, 1.14
Constant	3.24***	2.15, 4.33	2.78***	1.49, 4.06
R^2^	.20		.20	
Akaike information criterion	15,533.9		15,528.4	
Bayesian information criterion	15,668.0		15,674.1	

*Note*: Controlled for gender, Latina/o/x or Hispanic race-ethnicity, year in college, campus, family financial strain, food security, and mother’s education.

***p < .001.

As shown in Table 3 Model 2, the relationship between threat to family and anxiety was not moderated by political engagement because the interaction term between threat to family and political engagement was not statistically distinct from zero (b = −.04, 95% CI = −.23, .15). We estimated predicted anxiety score at four levels of political engagement to ease interpretation (never = 0, rarely = 1, sometimes = 2, often = 3) using the results from Model 2. At all levels of political engagement, threat to family was positively associated with anxiety, and the slopes of the individual lines did not vary (results not shown).

### Multivariate OLS Results by Self/Parental Status

Table 4 compares the main and interaction effects for adjusted regression results of threat to family on anxiety, stratified by self/parental status. In each of the main effects models, there is a positive and statistically significant effect of threat to family on anxiety, consistent with the aggregated results. Undocumented students had the largest coefficient (b = 1.72, 95% CI = 1.23 to 2.22), nearly double the coefficient for U.S. citizen students with lawfully present parents (b = 0.94, 95% CI = 0.61 to 1.27).

**Table 4.** Adjusted Ordinary Least Squares Regression Moderation Results for Threat to Family and Anxiety by Self/Parental Status Group, UC PromISE Survey (*N* = 2,511).

	U.S. Citizen Students with Lawfully Present Parents *n* = 1,293		U.S. Citizen Students with Undocumented Parents *n* = 609		Undocumented Students *n* = 609	
	Main	Interaction	Main	Interaction	Main	Interaction
Threat to family	.94***	1.49***	1.07***	.55	1.72***	1.20**
Political engagement		1.39***		−.92		−1.62
Threat to family × Political engagement		−.43*		.36		.44
Constant	3.18***	1.63	3.51	5.21	−3.59	−1.80

*Note*: Controlled for gender, Latina/o/x or Hispanic race-ethnicity, year in college, campus, family financial strain, food security, and mother’s education.

* *p* < .05, **p < .01, ***p < .001.

**Figure 2.**
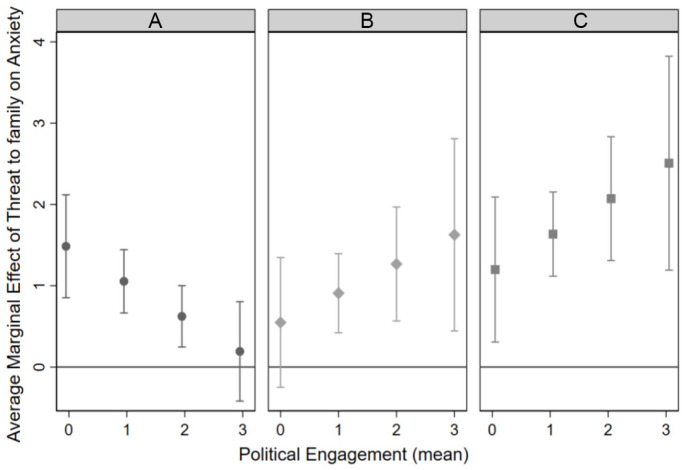
Average Marginal Effect of Threat to Family on Anxiety by Political Engagement Level and Self/Parental Status. (a) Citizens with Lawfully Present Parents. (b) Citizens with Undocumented Parents. (c) Undocumented Students. *Note*: Each graph depicts the interaction effect for each self/parental status group. Within each graph, each plot depicts the average effect of a one-unit change in threat to family on anxiety score with its corresponding 95% confidence interval. The average marginal effects were plotted at one-unit intervals between the minimum (0) and maximum (3) political engagement levels.

The interaction effects varied by group (see Figure 2; Appendix Figure C1 in the online version of the article). Appendix Figure C1 in the online version of the article shows the predicted means of threat to family on anxiety for students with the lowest (solid) and highest (long dash dot) levels of political engagement for each self/parental status group. Figure 2 shows the average marginal effects of a one-unit change in threat to family on anxiety score at four levels of political engagement (0, 1, 2, 3) for each self/parental status group.

Among U.S. citizen students with lawfully present parents, the effect of threat to family on anxiety was moderated by political engagement mean score (b = −.43, 95% CI = −0.78, −0.08). The negative interaction coefficient in Table 4 suggests that as political engagement increases, the relationship between threat to family and anxiety symptoms diminishes. As illustrated in Figure 2a, for students who have the lowest political engagement mean score (0), each additional unit of threat to family significantly increases anxiety scores by 1.49 on average (Appendix Table C1 in the online version of the article, dy/dx = 1.49, 95% CI = .85, 2.12). However, for students who have the highest political engagement mean score (3), the average marginal effect is not statistically significantly different from 0 (Appendix Table C1 in the online version of the article, dy/dx = .19, 95% CI = −.42, .80). In other words, among U.S. citizen students with lawfully present parents, threat to family was associated with more anxiety symptoms among those who were less politically engaged and not among those who were more politically engaged.

By contrast, we did not detect a moderating effect of political engagement among U.S. citizen students with undocumented parents or undocumented students. Among U.S. citizen students with undocumented parents, threat to family was positively associated with GAD-7 (b = 1.07; 95% CI = .61, 1.54). Figure 2b suggests a slight trend such that threat to family was associated with anxiety symptoms among more politically engaged (1–3) U.S. citizen students with undocumented parents and not those who are the least politically engaged (0; for average marginal effects, seeAppendix Table C1 in the online version of the article). However, we acknowledge that this effect is likely small, given the null interaction coefficient in the regression model (b = .36; 95% CI = −.21, .93) and the large confidence intervals at high levels of political engagement. Thus, the relationship between threat to family and anxiety symptoms does not vary significantly by political engagement level for U.S. citizen students with undocumented parents.

Among undocumented students, threat to family was positively associated with GAD-7 (b = 1.72, 95% CI = 1.23, 2.22), but there was no significant interaction between threat to family and political engagement on GAD-7 (b = .44, 95% CI = −0.21, 1.08). Threat to family was clearly associated with more anxiety symptoms at all levels of political engagement (Figure 2c). The absence of a moderation effect among U.S. citizen students with undocumented parents and undocumented students suggests that political engagement does not lessen anxiety among legally vulnerable students.

### Sensitivity Checks

We reran our stratified models with categorical coding for political participation and family threat to consider an alternate functional form between family threat and anxiety. For family threat, we categorized respondents into tertiles based on their group-specific distribution, and for political engagement, we compared those with no engagement versus others. These categorical models confirmed that as family threat increases, anxiety increases in a stepwise fashion and that political engagement reduces the differences in anxiety between those with high and low family threat—but for U.S. citizen students with lawfully present parents only (Online Appendix E in the online version of the article). The Wald tests further supported our earlier finding that moderation was detected only among students with lawfully present parents.

## DISCUSSION

This article aimed to advance research by exploring how the family immigration context—that is, immigration-related threat to family and family member’s immigration statuses—may affect individual members’ mental health. We analyzed the relationship between perceived threat to family due to the immigration policy context and anxiety symptomatology among immigrant-origin students, tested political engagement as a moderator, and stratified analyses by self/parental status to determine if different effects existed. We found threat to family to be positively associated with increased anxiety among all respondents, both in the aggregated sample and when stratified by self/parental immigration status. Among the aggregated sample, political engagement did not moderate the association between threat to family and anxiety, although the negative interaction coefficient suggested a potentially protective moderation effect at high levels of political engagement. In our stratified analyses, we found that political engagement moderated the relationship between threat to family and anxiety symptoms only among U.S. citizen students with lawfully present parents. These findings highlight activisms’ potential to offset the mental strain of immigration-related threats, yet its agentic effects do not seem to protect students who are most affected by legal vulnerability within their families.

Our study aligns with others who have found that exclusionary immigration policy negatively affects mental health (Hatzenbuehler et al. 2017; Philbin et al. 2018). More frequent perceptions of threat to family are associated with poorer mental health outcomes among undocumented students (Velarde Pierce et al. 2021), Latinx immigrant parents (Ayón 2020), and U.S.-born Latinx adolescents (Eskenazi et al. 2019). Our study extends this positive relationship between threat to family and anxiety to college students of various self/parent immigration statuses. It is especially noteworthy that U.S. citizen students with lawfully present parents demonstrated the same relationship as our other two student groups, underscoring the pervasive reach of immigration threats within immigrant-origin communities. The significant relationship for all three types of immigrant-origin students further illustrates the importance of family-based legal vulnerability as a structural determinant of health (Enriquez 2020; Montez et al. 2021). Prior research has focused on individual immigration status as a major risk factor for poorer mental health outcomes. By contrast, our findings highlight the shared risks within families determined by legal and political factors. Further research on immigrant health should prioritize understanding how differential advantage maintains inequities through a structural determinants lens (García et al. 2021; Misra et al. 2021).

It is important to consider that this study took place in California, one of the most inclusive state contexts for undocumented immigrants (Young and Wallace 2019). California provides access to drivers’ licenses and limits participation with federal immigration enforcement programs. Such policies curtail deportation threats and may mitigate threat to family. We found a positive association between threat to family and anxiety despite this inclusive context. Thus, our results are likely a conservative estimate of the impact of immigration-related threat to family on mental health, warranting future investigations in other locales.

Our findings also demonstrate that political engagement appears to moderate immigration-policy-related stressors to a limited degree. For politically engaged students, the association between threat to family and anxiety was attenuated. This protective effect aligns with nascent research that has found protective effects of activism for the mental health of students of color (Frost et al. 2019; Hope et al. 2018). Yet the moderating effect was statistically significant only for U.S. citizen students with lawfully present parents. It is possible that political engagement operated as expected among this group because they felt a greater sense of agency related to the immigration policy context and their political engagement. Given their own and their parents’ lawful presence, this group of students experienced less legal vulnerability within their immediate family context. Their political engagement would likely not have been curtailed by their own family members’ legal vulnerability, potentially increasing their feelings of agency to participate. Although their political engagement may or may not have been related to immigration policy, attempting to affect change, learning about political structures, and experiencing successful campaigns could have increased their feelings of agency in the face of structural inequality, weakening the effect that policy threats might have on their mental health. Furthermore, it is most likely that they perceived immigration-related threats for their extended family members, meaning that they may not have felt as severe a threat to their own everyday lives or well-being, weakening the relationship between threat to family and anxiety. They may have felt that their own and/or parents’ citizenship privilege would enable their agentic interventions to support their family members in the face of these threats.

Political engagement was not a significant protective factor for undocumented students or for U.S. citizen students with undocumented parents. These two groups of students may have stronger associations between threat to family and anxiety that could not be easily alleviated through political engagement. Students may sign a petition in support of releasing detainees or post #HereToStay on social media because they want to use their political voice (Muñoz 2016; Wilf, Castro, and Gupta 2022), but these actions would not immediately benefit their family’s safety and security in light of persistent policy threats. On the other hand, decisions to engage politically, especially in public forms of political engagement like protests, are perceived as risky for student and parent safety. Students have to decide whether the potential change in future material conditions prevails over the more immediate risk of disclosing their status and potentially putting themselves and their families at risk for deportation (Enriquez and Saguy 2016). Furthermore, political engagement “helps undocumented young people to feel like they are being proactive, but it does not take the fear away” (Nájera 2020:354). Thus, our findings suggest that the persistent legal vulnerability within their family is much stronger than any of the agentic benefits offered by political engagement.

It is plausible that political engagement might buffer against anxiety by increasing students’ social support. In fact, organization membership has been a significant determinant of political participation among undocumented students (Wong et al. 2019). It is possible that as immigrant-origin students join political organizations, they benefit from emotional sustenance, active coping assistance, or social influence (Thoits 2011). However, recent work evaluating dimensions of legal vulnerability on undocumented students’ mental health found that anxiety was not buffered by social support (Velarde Pierce et al. 2021). Furthermore, our conceptualization of political engagement included expressions of political voice, such as signing petitions or attending protests, which do not capture the community-building aspects of organizing. Additional research is needed to clarify whether and how social ties contribute to political engagement among immigrant-origin students.

### Limitations and Future Directions

Our study is of a nonprobability sample. We are unable to determine the representativeness of our sample due to the lack of institutional data that include self/parental immigration status. Yet UC serves as an ideal site to recruit a large sample given its location in the state with the largest population of undocumented students. Without a sampling frame, it is also possible that selection bias played a factor among our participants. That is, respondents may have been less worried about their families, less anxious, and/or more politically engaged than the immigrant-origin students who did not complete the survey. However, responses for key variables were reported across the entire range of possible answers (see Appendix B in the online version of the article). Because the distributions were not skewed, we believe that potential selection biases on these key variables were negligible. Beyond these sampling concerns, it is also plausible that students with fewer anxiety symptoms engage in more political activities. We could not quantitatively assess reverse causality with this cross-sectional survey.

Our study did not distinguish between different forms of political engagement. It is possible that specific forms of political engagement (i.e., active vs. passive political engagement) could be effective at reducing anxiety. Relatedly, the survey did not ask which social causes motivated respondents’ engagement. Follow-up interviews suggest that undocumented students and U.S. citizen students with undocumented parents focus on political issues directly affecting the immigrant community (Enriquez et al. Forthcoming-b). Yet engaging in any kind of political activism, even if not directly related to immigration, could reduce anxiety by raising one’s sense of control and agency (Thoits 2006). Future research should assess whether the modality of engagement and the specific cause for advocacy impact the extent to which students’ immigration-related stressors are alleviated.

It is also possible that political engagement behaviors reported by students were influenced by a period effect of increased public attention to racism. Although the racial reckoning that followed George Floyd’s murder on May 25, 2020, undoubtedly shaped young adults’ political activism, data collection concluded at the end of each campus’s spring 2020 term. Because campuses on the semester system ended mid-May and those on the quarter system ended early June, it is unlikely that the relationships observed in our analysis were driven primarily by these demonstrations. That said, non-Black immigrant-origin students who support the Movement for Black Lives may view their involvement as an expression of multiracial solidarity to counter the overpolicing of communities of color (Terriquez and Milkman 2021). Indeed, the recognition of distinct yet shared struggles is critical for addressing racial health equity.

### Policy and Practice Implications

Collegiate mental health tends to focus on individual behaviors, like seeking therapy, rather than addressing the fundamental causes of injustice. As is the case for immigrant mental health, the context that shapes stressors can no longer be ignored (Takeuchi 2016). We recommend that researchers and practitioners expand conventional notions of treatment to advocate for safer communities for undocumented students, leveraging university policies when possible (Enriquez et al. 2019; French et al. 2020; Suárez-Orozco et al. 2015). Given the heavy toll that activism can take on legally vulnerable student activists’ mental health, wellness strategies should include working toward equitable allocation of emotional, financial, and legal support for undocumented students and students with undocumented parents.

To improve the mental well-being of immigrant-origin students, we must also address the roots of oppression for immigrant communities (García et al. 2021). Policies curtailing local immigration enforcement activities would likely reduce anxiety by lowering perceptions of threat to family (Flores Morales and Garcia 2021; Valdivia 2019). A longer-term solution would require the creation of a pathway to citizenship for all undocumented immigrants rather than limited protections offered to a small segment of the population. Because engaging politically appears to be beneficial for U.S. citizen students with lawfully present parents, advantaged groups should acknowledge their privilege and exercise their political voice to demand immigrant justice.

## CONCLUSION

Our study provides insight into the negative mental health implications of restrictive immigration policies that threaten family security and stability. By focusing on immigration-related threat to family, we call attention to the family-level implications of such policies. By including students with various self/parental immigration statuses, we expand the understanding of who is impacted by immigration policy beyond undocumented students to include U.S. citizen students with undocumented parents and even U.S. citizen students with lawfully present parents. This not only expands the scope of the stakes but also highlights the commonalities and potential for cultivating solidarity across various immigration statuses. At the nexus of immigration policy, college student mental health, and sociopolitical development, this study has implications for research, policy, and practice regarding structural determinants of health, medical sociology, and coping mechanisms.

## Supplemental Material

sj-docx-1-hsb-10.1177_00221465241247541 – Supplemental material for Anxious Activists? Examining Immigration Policy Threat, Political Engagement, and Anxiety among College Students with Different Self/Parental Immigration Statuses Supplemental material, sj-docx-1-hsb-10.1177_00221465241247541 for Anxious Activists? Examining Immigration Policy Threat, Political Engagement, and Anxiety among College Students with Different Self/Parental Immigration Statuses by Erin Manalo-Pedro, Laura E. Enriquez, Jennifer R. Nájera and Annie Ro in Journal of Health and Social Behavior
