# Supplementary material for: Anxious Activists? Examining Immigration Policy Threat, Political Engagement, and Anxiety among College Students with Different Self/Parental Immigration Statuses
Source: J Health Soc Behav. 2024 Apr 29;65(3):381–99. doi: 10.1177/00221465241247541 (PMC11380356; doi:10.1177/00221465241247541)

**Journal** of **Health**

and **Social Behavior**

OFFICIAL JOURNAL OF THE AMERICAN SOCIOLOGICAL ASSOCIATION

**ONLINE SUPPLEMENT**

**to article in**

Journal of Health and Social Behavior

**Anxious Activists? Examining Immigration Policy Threat, Political Engagement, and Anxiety Among College Students with Different Self/Parental Immigration Statuses**

**Erin Manalo-Pedro**

*University of California, Los Angeles*

**Laura E. Enriquez**

*University of California, Irvine*

**Jennifer R. Nájera**

*University of California, Riverside*

**Annie Ro**

*University of California, Irvine*

# Appendix A. Comparison of anxiety means by survey timing

Table A1. Anxiety mean scores before and after COVID questions were added

|  | BEFORE additional COVID questions | AFTER additional COVID questions | Total |
| --- | --- | --- | --- |
|  | N=394 | N=2117 | N=2511 |
| Generalized anxiety disorder 7-item (GAD-7) total | 8.54 (5.9) | 8.67 (5.9) | 8.65 (5.9) |

Table A2. Two-sample t-test for equality of means

|  | t | df | Sig. (2-tailed) | Mean difference | SE | 95% CI |
| --- | --- | --- | --- | --- | --- | --- |
| Generalized anxiety disorder 7-item (GAD-7) total | 0.3973 | 2509 | 0.691 | 0.129 | 0.324 | (-0.51, 0.76) |

# Appendix B. Key Variable Items by Self/Parental Status

Table B1. Anxiety, threat to family, and political engagement items by self/parental status, UC PromISE Survey (n = 2,511)

|  | U.S. Citizen Students with Lawfully Present Parents | U.S. Citizen Students with Undocumented Parents | Undocumented Students | Total | p-value |
| --- | --- | --- | --- | --- | --- |
|  | N=1,293 | N=609 | N=609 | N=2,511 |  |
| *Generalized anxiety disorder (GAD-7)* | | | | | |
| Feeling nervous, anxious, or on edge |  |  |  |  | ** |
| Not at all | 263 (20.3%) | 93 (15.3%) | 105 (17.2%) | 461 (18.4%) |  |
| Several days | 510 (39.4%) | 231 (37.9%) | 223 (36.6%) | 964 (38.4%) |  |
| More than half the days | 308 (23.8%) | 143 (23.5%) | 165 (27.1%) | 616 (24.5%) |  |
| Nearly every day | 212 (16.4%) | 142 (23.3%) | 116 (19.0%) | 470 (18.7%) |  |
| Not being able to stop or control worrying |  |  |  |  |  |
| Not at all | 397 (30.7%) | 172 (28.2%) | 158 (25.9%) | 727 (29.0%) |  |
| Several days | 421 (32.6%) | 186 (30.5%) | 208 (34.2%) | 815 (32.5%) |  |
| More than half the days | 277 (21.4%) | 135 (22.2%) | 137 (22.5%) | 549 (21.9%) |  |
| Nearly every day | 198 (15.3%) | 116 (19.0%) | 106 (17.4%) | 420 (16.7%) |  |
| Worrying too much about different things |  |  |  |  |  |
| Not at all | 254 (19.6%) | 95 (15.6%) | 90 (14.8%) | 439 (17.5%) | ** |
| Several days | 482 (37.3%) | 204 (33.5%) | 220 (36.1%) | 906 (36.1%) |  |
| More than half the days | 307 (23.7%) | 156 (25.6%) | 150 (24.6%) | 613 (24.4%) |  |
| Nearly every day | 250 (19.3%) | 154 (25.3%) | 149 (24.5%) | 553 (22.0%) |  |
| Trouble relaxing |  |  |  |  |  |
| Not at all | 354 (27.4%) | 146 (24.0%) | 127 (20.9%) | 627 (25.0%) | ** |
| Several days | 482 (37.3%) | 201 (33.0%) | 223 (36.6%) | 906 (36.1%) |  |
| More than half the days | 249 (19.3%) | 133 (21.8%) | 140 (23.0%) | 522 (20.8%) |  |
| Nearly every day | 208 (16.1%) | 129 (21.2%) | 119 (19.5%) | 456 (18.2%) |  |
| Being so restless that it is hard to sit still |  |  |  |  |  |
| Not at all | 682 (52.7%) | 287 (47.1%) | 294 (48.3%) | 1263 (50.3%) |  |
| Several days | 357 (27.6%) | 185 (30.4%) | 168 (27.6%) | 710 (28.3%) |  |
| More than half the days | 139 (10.8%) | 75 (12.3%) | 85 (14.0%) | 299 (11.9%) |  |
| Nearly every day | 115 (8.9%) | 62 (10.2%) | 62 (10.2%) | 239 (9.5%) |  |
| Becoming easily annoyed or irritable |  |  |  |  | * |
| Not at all | 346 (26.8%) | 148 (24.3%) | 122 (20.0%) | 616 (24.5%) |  |
| Several days | 512 (39.6%) | 236 (38.8%) | 244 (40.1%) | 992 (39.5%) |  |
| More than half the days | 268 (20.7%) | 128 (21.0%) | 136 (22.3%) | 532 (21.2%) |  |
| Nearly every day | 167 (12.9%) | 97 (15.9%) | 107 (17.6%) | 371 (14.8%) |  |
| Feeling afraid, as if something awful might happen |  |  |  |  | *** |
| Not at all | 560 (43.3%) | 211 (34.6%) | 199 (32.7%) | 970 (38.6%) |  |
| Several days | 395 (30.5%) | 194 (31.9%) | 200 (32.8%) | 789 (31.4%) |  |
| More than half the days | 186 (14.4%) | 113 (18.6%) | 110 (18.1%) | 409 (16.3%) |  |
| Nearly every day | 152 (11.8%) | 91 (14.9%) | 100 (16.4%) | 343 (13.7%) |  |
| *Threat to Family* | | | | | |
| Do you worry about the impact immigration policies have on you or your family? |  |  |  |  | *** |
| Never | 471 (36.4%) | 29 (4.8%) | 14 (2.3%) | 514 (20.5%) |  |
| Rarely | 215 (16.6%) | 14 (2.3%) | 4 (0.7%) | 233 (9.3%) |  |
| Sometimes | 356 (27.5%) | 90 (14.8%) | 58 (9.5%) | 504 (20.1%) |  |
| Often | 147 (11.4%) | 137 (22.5%) | 159 (26.1%) | 443 (17.6%) |  |
| Always | 104 (8.0%) | 339 (55.7%) | 374 (61.4%) | 817 (32.5%) |  |
| Do you fear that you or a family member will be reported to immigration officials? |  |  |  |  | *** |
| Never | 805 (62.3%) | 28 (4.6%) | 33 (5.4%) | 866 (34.5%) |  |
| Rarely | 185 (14.3%) | 61 (10.0%) | 74 (12.2%) | 320 (12.7%) |  |
| Sometimes | 195 (15.1%) | 134 (22.0%) | 166 (27.3%) | 495 (19.7%) |  |
| Often | 62 (4.8%) | 147 (24.1%) | 122 (20.0%) | 331 (13.2%) |  |
| Always | 46 (3.6%) | 239 (39.2%) | 214 (35.1%) | 499 (19.9%) |  |
| Do you worry about family separation due to deportation? |  |  |  |  | *** |
| Never | 756 (58.5%) | 16 (2.6%) | 23 (3.8%) | 795 (31.7%) |  |
| Rarely | 195 (15.1%) | 26 (4.3%) | 41 (6.7%) | 262 (10.4%) |  |
| Sometimes | 189 (14.6%) | 100 (16.4%) | 95 (15.6%) | 384 (15.3%) |  |
| Often | 69 (5.3%) | 112 (18.4%) | 119 (19.5%) | 300 (11.9%) |  |
| Always | 84 (6.5%) | 355 (58.3%) | 331 (54.4%) | 770 (30.7%) |  |
| *Political Engagement* | | | | | |
| Contact a public official - at any level of government - to express your opinion |  |  |  |  | * |
| Never | 885 (68.7%) | 371 (61.2%) | 411 (67.8%) | 1667 (66.7%) |  |
| Rarely | 256 (19.9%) | 142 (23.4%) | 135 (22.3%) | 533 (21.3%) |  |
| Sometimes | 119 (9.2%) | 71 (11.7%) | 46 (7.6%) | 236 (9.4%) |  |
| Often | 29 (2.2%) | 22 (3.6%) | 14 (2.3%) | 65 (2.6%) |  |
| Take part in a protest, march, or demonstration, or rally on-campus |  |  |  |  | *** |
| Never | 625 (48.5%) | 204 (33.5%) | 235 (38.8%) | 1064 (42.5%) |  |
| Rarely | 250 (19.4%) | 133 (21.8%) | 130 (21.5%) | 513 (20.5%) |  |
| Sometimes | 277 (21.5%) | 158 (25.9%) | 162 (26.8%) | 597 (23.9%) |  |
| Often | 136 (10.6%) | 114 (18.7%) | 78 (12.9%) | 328 (13.1%) |  |
| Take part in a protest, march, or demonstration, or rally off-campus |  |  |  |  | *** |
| Never | 726 (56.5%) | 259 (42.5%) | 291 (47.9%) | 1276 (51.0%) |  |
| Rarely | 268 (20.8%) | 129 (21.2%) | 143 (23.5%) | 540 (21.6%) |  |
| Sometimes | 219 (17.0%) | 148 (24.3%) | 110 (18.1%) | 477 (19.1%) |  |
| Often | 73 (5.7%) | 73 (12.0%) | 64 (10.5%) | 210 (8.4%) |  |
| Sign a petition regarding an issue or problem that concerns you |  |  |  |  | *** |
| Never | 134 (10.5%) | 42 (7.0%) | 113 (18.9%) | 289 (11.7%) |  |
| Rarely | 209 (16.4%) | 69 (11.5%) | 107 (17.9%) | 385 (15.5%) |  |
| Sometimes | 485 (37.9%) | 201 (33.6%) | 192 (32.1%) | 878 (35.5%) |  |
| Often | 450 (35.2%) | 287 (47.9%) | 187 (31.2%) | 924 (37.3%) |  |
| Boycott a company or product for social or political reasons |  |  |  |  | * |
| Never | 419 (32.9%) | 199 (32.8%) | 201 (33.3%) | 819 (33.0%) |  |
| Rarely | 254 (19.9%) | 95 (15.7%) | 105 (17.4%) | 454 (18.3%) |  |
| Sometimes | 326 (25.6%) | 137 (22.6%) | 148 (24.5%) | 611 (24.6%) |  |
| Often | 275 (21.6%) | 175 (28.9%) | 149 (24.7%) | 599 (24.1%) |  |
| Buy a certain product or service because you like the social or political values of the company |  |  |  |  |  |
| Never | 339 (26.5%) | 135 (22.3%) | 153 (25.5%) | 627 (25.2%) |  |
| Rarely | 201 (15.7%) | 94 (15.5%) | 98 (16.4%) | 393 (15.8%) |  |
| Sometimes | 377 (29.5%) | 175 (28.9%) | 175 (29.2%) | 727 (29.3%) |  |
| Often | 363 (28.4%) | 201 (33.2%) | 173 (28.9%) | 737 (29.7%) |  |
| Discuss political issues on social media |  |  |  |  | *** |
| Never | 509 (39.6%) | 204 (33.8%) | 205 (33.9%) | 918 (36.8%) |  |
| Rarely | 263 (20.5%) | 103 (17.1%) | 126 (20.9%) | 492 (19.7%) |  |
| Sometimes | 267 (20.8%) | 118 (19.5%) | 128 (21.2%) | 513 (20.6%) |  |
| Often | 247 (19.2%) | 179 (29.6%) | 145 (24.0%) | 571 (22.9%) |  |
| Wear buttons or display stickers with social or political messages |  |  |  |  | *** |
| Never | 579 (45.0%) | 200 (33.2%) | 243 (40.0%) | 1022 (41.0%) |  |
| Rarely | 269 (20.9%) | 114 (18.9%) | 113 (18.6%) | 496 (19.9%) |  |
| Sometimes | 235 (18.3%) | 117 (19.4%) | 111 (18.3%) | 463 (18.6%) |  |
| Often | 203 (15.8%) | 171 (28.4%) | 140 (23.1%) | 514 (20.6%) |  |
| Expressed a political point of view during a class discussion |  |  |  |  | * |
| Never | 390 (30.5%) | 154 (25.6%) | 159 (26.3%) | 703 (28.3%) |  |
| Rarely | 265 (20.7%) | 122 (20.3%) | 131 (21.7%) | 518 (20.8%) |  |
| Sometimes | 336 (26.3%) | 146 (24.3%) | 172 (28.5%) | 654 (26.3%) |  |
| Often | 288 (22.5%) | 180 (29.9%) | 142 (23.5%) | 610 (24.5%) |  |

Notes: The following prompt preceded the GAD-7 items, “Over the last two weeks, how often have you been bothered by the following problems?” The following prompt preceded the political engagement items, “Below is a list of things that some people do to express their views. For each one, identify how often you do it.” The following p-values correspond to chi-squared test of independence for categorical variables: * p < 0.05, ** p < 0.01, *** p < 0.001.

# Appendix C. Marginal Effects – Stratified Analysis

Table C1. Average marginal effects of threat to family on anxiety by political engagement level and self/parental status, UC PromISE Survey (n = 2,511)

|  | Average change in anxiety / threat to family | Delta-method |  |  |  |  |
| --- | --- | --- | --- | --- | --- | --- |
| Political engagement level | dy/dx | Std. Err. | t | P>\|t\| | [95% CI] | |
| U.S. citizen students with lawfully present parents |  |  |  |  |  |  |
| 0 | 1.49 | .32 | 4.60 | 0.000 | .85 | 2.12 |
| 1 | 1.05 | .20 | 5.32 | 0.000 | .67 | 1.44 |
| 2 | .62 | .19 | 3.24 | 0.001 | .25 | 1.00 |
| 3 | .19 | .31 | 0.62 | 0.537 | -.42 | .80 |
| U.S. citizen students with undocumented parents |  |  |  |  |  |  |
| 0 | .55 | .41 | 1.35 | 0.177 | -.25 | 1.35 |
| 1 | .91 | .25 | 3.66 | 0.000 | .42 | 1.40 |
| 2 | 1.27 | .36 | 3.55 | 0.000 | .57 | 1.97 |
| 3 | 1.63 | .60 | 2.70 | 0.007 | .44 | 2.81 |
| Undocumented students |  |  |  |  |  |  |
| 0 | 1.20 | .45 | 2.64 | 0.008 | .31 | 2.09 |
| 1 | 1.64 | .26 | 6.20 | 0.000 | 1.12 | 2.15 |
| 2 | 2.07 | .39 | 5.34 | 0.000 | 1.31 | 2.83 |
| 3 | 2.51 | .67 | 3.74 | 0.000 | 1.19 | 3.82 |

Figure C1. Predictive margins of threat to family on anxiety by political engagement level and self/parental status


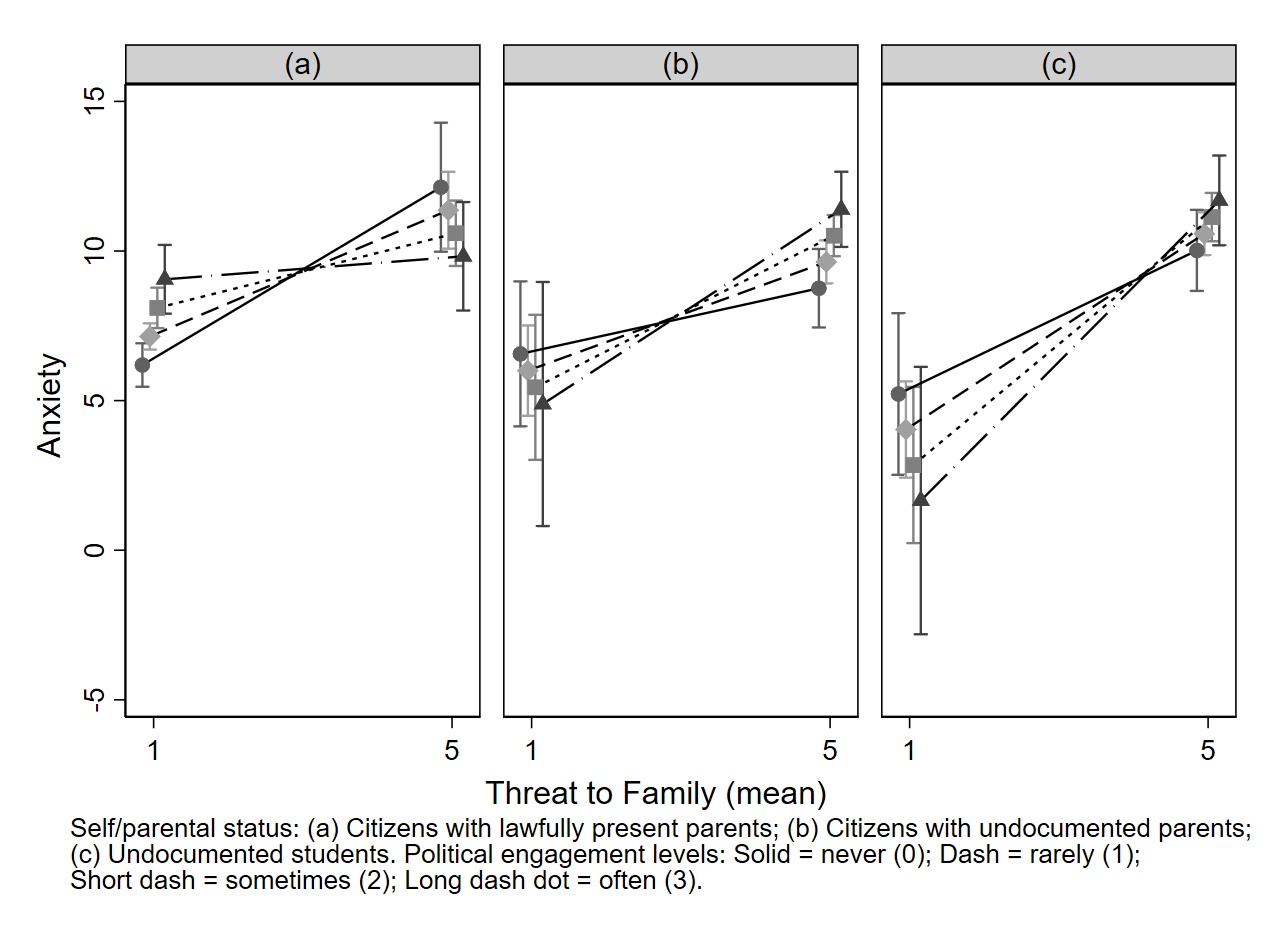


# Appendix D. Three-Way Interaction Analysis (OLS Regression and Marginal Effects)

Table D1. Adjusted OLS regression results on anxiety, three-way interaction for threat to family, political engagement, and self/parental status group, UC PromISE Survey (n = 2,511)

| Variable | Model 4 | | Model 5 | | Model 6 | |
| --- | --- | --- | --- | --- | --- | --- |
|  | Coef. | 95% CI | Coef. | 95% CI | Coef. | 95% CI |
| Threat to family | 0.67*** | 0.36-0.99 | 1.10*** | 0.75-1.45 | 1.49*** | 0.87-2.12 |
| Political engagement | 0.68* | 0.02-1.34 | 0.57 | -0.09 -1.23 | 1.35** | 0.54-2.15 |
| Threat to family x PE | -0.02 | -0.21 - 0.17 | -0.04 | -0.23 -0.15 | -0.44* | -0.79 - -0.09 |
| Self/parental status |  |  |  |  |  |  |
| U.S. citizen students with lawfully present parents |  |  | Ref |  | Ref |  |
| U.S. citizen students with undocumented parents |  |  | -1.77*** | -2.47 - -1.07 | 0.21 | -3.19 - 3.61 |
| Undocumented students |  |  | -1.82*** | -2.52 - -1.13 | -0.66 | -4.18 - 2.85 |
| Self/parental status x Threat to family |  |  |  |  |  |  |
| U.S. citizen students with lawfully present parents |  |  |  |  | Ref |  |
| U.S. citizen students with undocumented parents |  |  |  |  | -0.86 | -1.87 - 0.15 |
| Undocumented students |  |  |  |  | -0.48 | -1.52 - 0.55 |
| Self/parental status x PE |  |  |  |  |  |  |
| U.S. citizen students with lawfully present parents |  |  |  |  | Ref |  |
| U.S. citizen students with undocumented parents |  |  |  |  | -2.17 | -4.75 - 0.41 |
| Undocumented students |  |  |  |  | -3.23* | -5.98 - -0.48 |
| Self/parental status x Threat to family x PE |  |  |  |  |  |  |
| U.S. citizen students with lawfully present parents |  |  |  |  | Ref |  |
| U.S. citizen students with undocumented parents |  |  |  |  | 0.81* | 0.14-1.48 |
| Undocumented students |  |  |  |  | 0.94* | 0.22-1.65 |
| Constant | 3.51*** | 2.05-4.97 | 3.12*** | 1.65-4.58 | 2.44** | 0.82-4.07 |
| R^2^ | 0.19 |  | 0.20 |  | 0.21 |  |
| AIC | 15561.0 |  | 15533.1 |  | 15527.3 |  |
| BIC | 15712.5 |  | 15696.3 |  | 15725.5 |  |

Notes: Controlled for gender; Latina/o/x or Hispanic race/ethnicity; year in college; campus; family financial strain; food security; and mother's education. PE = Political Engagement.

Table D2. Average marginal effects of threat to family on anxiety by political engagement level and self/parental status, UC PromISE Survey (n = 2,511)

|  | Average change in anxiety / threat to family | Delta-method |  |  |  |  |
| --- | --- | --- | --- | --- | --- | --- |
| Political engagement level | dy/dx | Std. Err. | t | P>\|t\| | [95% CI] | |
| U.S. citizen students with lawfully present parents |  |  |  |  |  |  |
| 0 | 1.49 | 0.32 | 4.71 | 0.000 | 0.87 | 2.12 |
| 1 | 1.05 | 0.19 | 5.52 | 0.000 | 0.68 | 1.43 |
| 2 | 0.62 | 0.19 | 3.29 | 0.001 | 0.25 | 0.98 |
| 3 | 0.18 | 0.31 | 0.57 | 0.570 | -0.43 | 0.78 |
| U.S. citizen students with undocumented parents |  |  |  |  |  |  |
| 0 | 0.63 | 0.41 | 1.55 | 0.120 | -0.16 | 1.43 |
| 1 | 1.00 | 0.24 | 4.14 | 0.000 | 0.53 | 1.47 |
| 2 | 1.37 | 0.35 | 3.90 | 0.000 | 0.68 | 2.06 |
| 3 | 1.74 | 0.60 | 2.90 | 0.004 | 0.56 | 2.91 |
| Undocumented students |  |  |  |  |  |  |
| 0 | 1.01 | 0.43 | 2.35 | 0.019 | 0.17 | 1.85 |
| 1 | 1.51 | 0.25 | 6.09 | 0.000 | 1.02 | 1.99 |
| 2 | 2.01 | 0.38 | 5.35 | 0.000 | 1.27 | 2.74 |
| 3 | 2.50 | 0.65 | 3.85 | 0.000 | 1.23 | 3.78 |

Figure D1. Average marginal effects of threat to family on anxiety by political engagement level and self/parental status, UC PromISE Survey (n = 2,511)


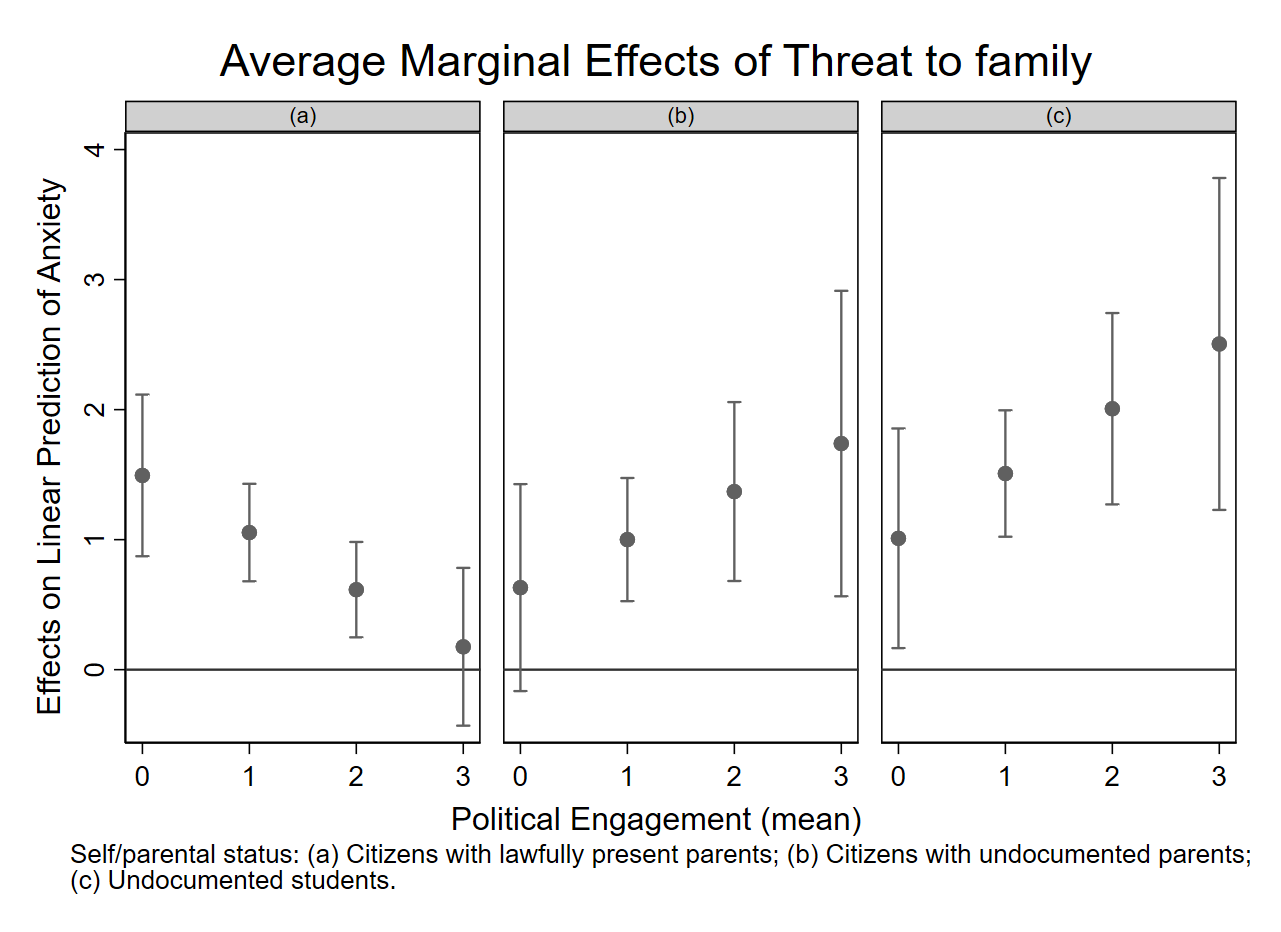


Note: These graphs illustrate the three-way interaction effect of threat to family, political engagement, and self/parental status group. Within each graph, each plot depicts the average effect of a 1-unit change in threat to family on anxiety score with its corresponding 95% confidence interval. The average marginal effects were plotted at 1-unit intervals between the minimum (0) and maximum (3) political engagement levels.

# Appendix E. Stratified Moderation Analysis with Categorical Variables (Marginal Effects)

Figure E1. Predictive margins of threat to family (categorical) on anxiety by political engagement level (none vs. any), stratified by self/parental status, UC PromISE Survey (n = 2,511)


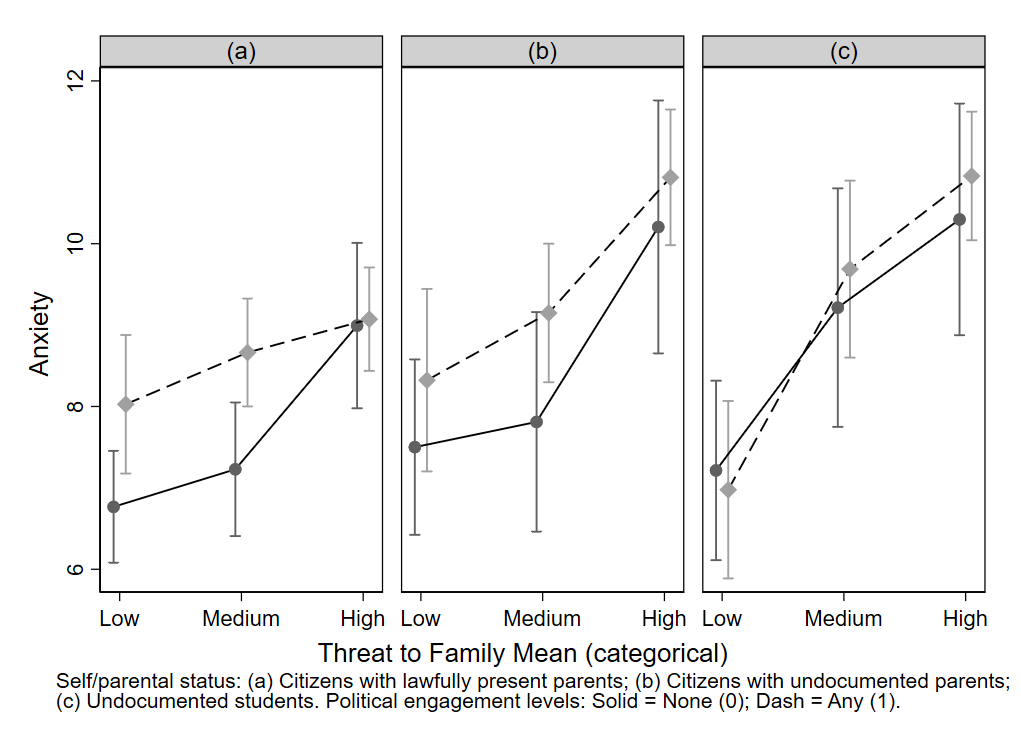

Supplement: sj-docx-1-hsb-10.1177_00221465241247541 – Supplemental material for Anxious Activists? Examining Immigration Policy Threat, Political Engagement, and Anxiety among College Students with Different Self/Parental Immigration Statuses [file sj-docx-1-hsb-10.1177_00221465241247541.docx]
